# Indents

**Published:** 1920-11

**Authors:** 


### INDENTS

**☞Brief Articles of Technical or Scientific Value will receive notice and comment. Contributions invited.**

---

CURETAGE. Next to prevention, curetage is probably the most important word in the vocabularly of modern dentistry. Whether it be removal of debris in caries, scaling pyorrhea pockets, or instrumentation following extraction of infected roots, thorough curetment is absolutely essential to the ultimate success of the operation.

---

WHAT SUPREME COURT DECISION IN OREGON MEANS. The opinion handed down recently by the Supreme Court of Oregon, will say that it is one of the most far reaching of its kind ever given by a court, in its bearing upon the dental profession. The dental profession of Oregon, as well as the general public, won a decisive victory over that element which is continually seeking, by hook or crook, to gain admission to the profession, irrespective of any qualifications as to fitness, by trainng along elementary principles, to engage in the legal practice of dentistry in Oregon.

The decision of the Supreme Court lays special emphasis on the fact that those who are entitled to a permit to maintain a dental office, must not do any act of dentistry, and clearly sets forth that all phases of the practice of dentistry must be done by properly licensed dentists. In effect, it means that any one who makes an examination of patients, or advises them in relation to their treatment, or even pulls or plugs a tooth, or manufactures or helps to manufacture false teeth for patients, without a license to practice dentistry, is a violator of the law against practicing dentistry and is subject to the provided penalties. This can mean nothing else but that office assistants, laboratory men, dental laboratories, X-ray laboratories, instructors in dental schools, or any other institution where any act of dentistry is performed, must be properly licensed persons. The opinion of the court upholds the contentions of the Board of Dental Examiners in every particular and it serves as a notice to all those who have not qualified to engage in the legal practice of dentistry, and to those that seek to legalize the so-called dental nurse to the practice of dentistry, that the present standards for admission to the legal practice of dentistry in Oregon has been definitely and decisively approved by thte highest tribunal of Oregon.—Board of Dental Examiners, H. H. Schmitt.

---

EATING TO LIVE. According to some authoritative writers whose opinions upon health and disease carry weight with the general public, the chief cause of dental disease and defective jaw development is a positively known and settled thing. Dr. Leonard Williams, in an article published last month in a London evening paper, states that the assumption that foods should be cooked, upon which modern theories of dietetics have been based, is entirely unwarranted. He stresses the vital and all-sufficient importance of eating raw foods, and concludes his article as follows: "If vitamine-containing, that is, raw foods are given in sufficient abundance there will be little trouble with the modern diseases above mentioned, and none with dental disease. The way to get rid of dental troubles in the young is to treat the children as we treat puppy dogs, by giving them something they must chew, and make them, in fact, work for their living."—*Dental Digest.*

---

A SHORT METHOD OF PLATE POLISHING. I desire to make a short addition to my article which appeared in the *Dental Digest* of March under the above caption. That article described a method of smoothing the wax baseplate by use of brush wheel, and coal-oil, so as to render the scraping and sand-papering of the vulcanite plate unnecessary. The addition is this: To prevent the adhesion of the plaster investment to the vulcanite, which is especially tenacious when the flask has been left unopened a long time after vulcaniaztion, work as follows: After removing the wax, if the plaster is very wet, dry it some, then bathe its surface with a thick coating of liquid silex for about five minutes, after which wash away the silex by a stream of water. The silex will wash away entirely from the teeth, but enough will remain soaked into the plaster to prevent its adhesion to the vulcanite. A similar use of silex applied to the cast (once known as "model") makes a fairly good substitute for tinfoil, but requires for its application the opening of the flask after its closure on the rubber. Unless I am mistaken, this method of using liquid silex originated with R. W. Tench, of New York.—Stewart J. Spence.

---

A Sectional Tray for Partial Impressions. Take an impression of the parts with modeling compound, placed in an oiled metal tray in the usual way; chill and remove from the mouth. Remove the compound from the tray and enlarge its impression side, cutting out all undercuts, so that when the compound is returned to the mouth it will pass in and out very easily. Roughen the impression side well, so that when the plaster is applied it will adhere to it. Now on the side of the compound which was next to the tray make a groove (from one end to the other) quite deep, then with a knife split it in two, three or four sections, as the case may demand. Put the fragments together and fasten them with beeswax. We now have a tray in sections which are fastened with wax and ready for the plaster. Mix the plaster thin, apply to the tray, and place in the mouth. When hard, the wax fastening the sections is scraped off and a knife is placed in the grooves and the sections plied apart. The impression usually comes off in as many sections as there are in the tray. —A. Kassab, in *Cosmos*.

THE CAST CLASP. Five years' experience with the cast clasp as devised by your essayist has brought out the following points:

That it is a most useful attachment for posterior removable bridges where mutilation of tooth structure is to be avoided.

That it will succeed in bridge cases where other types of clasps have failed.

That it is adaptable to all variations of form of bicuspids and molars, and over ninety per cent. of cuspids.

That it is practically immune to breakage providing a suitable gold alloy is used for casting.

That the abutment teeth and adjacent tissues remain in good condition.

That patients *will* remove and clean the appliance if *properly instructed* and due emphasis is placed on the *importance* of such cleaning.

That the finished clasp is no better than the wax pattern.

That the correct designing of the wax pattern has largely to do with the success of the finished bridge.

That the cast clasp is useful only *in a very limited number* of cases of *partial plates*.

That a method, although simple, may not be as easy as it seems.—N. B. Nesbit, in *Cosmos*.

---

TREATMENT OF NON-VITAL TEETH. (1) As devitalized teeth are the cause of most pathological areas, our dental treatment should be principally confined to one definite purpose; that is, to the maintenance of vital healthy pulps in our teeth. There can be no better root canal filling.

(2) As root canal work is necessary under our present civilization, it should be obligatory on us to check our work by roentgenograms when possible. Root canals can be perfectly filled * * * sometimes.

(3) Root canals should not be filled with radiolucent material.

(4) Pathological areas may or may not contain pyogenic bacteria, and as we are unable, at present, to differentiate between septic and non-septic areas, either clinically or roentgenographically, we are compelled to classify all such as septic—or likely to become septic—especially in patients suffering from any form of toxæmia.

(5) It is for us, as dentists, to carry out this reform as a *modus operandi* of our work, and not to have it forced on us by medical practitioners or suffering patients; thus shall we bring dentistry into line with the other branches of the healing art and command the respect of the medical profession.—H. C. Moxham, in *Commonwealth Dental Review.*

---

WHY NOT BE SANE? This query is raised as the result of a long observation of men and their methods, not only in the practice of dentistry, but in medicine as well. The intimation is, of course, not made that dentists and medical men are insane—far from it—but the fact is that in some sense, and at certain times, professional men seem to have a tendency to run off so far at a tangent as to encourage the inference that they were lacking in mental balance. Take the various fads which have from time to time ridden rough-shod over the common sense of dentists. To enumerate only a few we may cite copper amalgam, cataphoresis, the "New Departure Creed," emetin for pyorrhea, analgesia, hypnotism, and more recently focal infection. The moment this latter is placed in the category of a fad we can hear a cry of holy horror go up from one end of the land to the other. The sacred precincts of a sublime truth are being invaded according to the notions of some of our very good men, and in this age a man must have much temerity to question the far-reaching effects of focal infection. Nor is it intended here to minimize the fact of focal infection, or its baneful influence under certain conditions, but merely to point out that it has been given undue significance in our literature, and particularly that the teeth as a causative factor in systemic disease have been exaggerated out of all proportion to their real merits. We have been given a mental bias over this question which has resulted in untold harm.

Why can not we be sane when it comes to judging the merits or demerits of any theory? Why must we constantly go to extremes? Why must poor humanity suffer so grievously because of our lack of mental balance? We do not seem to profit one iota by the blunders which we have made in the past. We go on permitting every hobby to take the bit in its teeth and carry us away beyond the goal.

The argument is often advanced that much good results from fads, and this in a certain sense is true. Without the enthusiasm which comes from following a fad there would not be much real progress, and this is asknowledged freely and frankly. Not only this, but in nearly every one of the fads which have hit dentistry, there was something of real merit—something which at the time entitled them to consideration.

But acknowledging all of this the fact remains that in a survey of the extremes to which the profession has gone it will be found that vastly more harm than good has been done, and in the ultimate it would have been better if the fad had never come.

Above all men on earth it would seem that the professional man was the one who should exhibit balance and clarity of reasoning. So much depends on his judgment in the critical issues of life and death that he should cultivate sanity and aim to preserve his equilibrium under all kinds of pressure. But instead of this he seems the first to be deflected by every passing whim, and to lead his patients into the highways and byways of experimentation. Men will make mistakes—it is inevitable—but they need not go on making the same mistake over and over again, to the serious injury of those whom they serve. The present plea is for breadth of vision, and greater sanity in our methods. —C. N. Johnson, in *Oral Health*.

---

Pathologic Conditions Under Fixed Bridgework. At this time, when the relative merits of fixed and removable bridgework are conspicuously on the *tapis,* it may be fitting to describe certain pathologic conditions which we have observed in the soft and calcified tissues immediately under and adjacent to pieces of fixed bridgework. In the *Pacific Dental Gazette* for May, 1916, Dr. James D. McCoy, in collaboration with the writer, in an article under the caption "Is Crown and Bridgework a Menace?" discussed the physiologic and pathologic aspects of fixed bridgework. The observations then recorded were based upon the study of a series of cases from private and infirmary practice. The reaction to mechanical and bacterial irritation, practically unavoidable in connection with fixed bridgework, was fully considered; pulp extirpation and the outcome of root canal operations were discussed from all angles bearing on the question, and finally, as the last proposition in the said communication, we alluded to fixation of the alveolo-dental articulation and to the disastrous results which follow such a procedure. We stated that atrophic changes in the peri-cemental fibers follow the fixation of the alveolo-dental articulation by bridgework of the permanent type. The peridental membrane, through the agency of the intra-alveolar fibers exhibits among many other functions that of enabling the tooth to have a certain degree of play in its socket, in the course of masticatory movements. In other words, in addition to acting as the retaining medium of the tooth in its alveolus, the fibers are almost constantly functionating, displaying a degree of elasticity which affords a range of interrupted movement to the tooth without which the blood vessels of the investing tissues would be deprived of a necessary physiologic stimulus. Because of these facts, and, furthermore, for the reason that any degree of fixation, which is another way of expressing functional inhibition, brings about corresponding degrees of degenerative or atrophic changes, fixed bridgework leads to changes of this character in the fibers of the peri-dental membrane, which last either completely disappear or become weak links in the chain of resistance to bacterial invasion.

In recent months we have occupied ourselves with radiographic studies of the soft and calcified tissues in relation with fixed bridgework. The conditions which the radiograms reveal have been found to be present in all cases in which the dummies were in close apposition with the mucous membrane; they were not found in the case of the so-called sanitary bridge in which an open space exists between the occlusal surface and the mucous membrane and from which porcelain facings are omitted. The lesion which was found in the osseous structure, as revealed by the radiogram, was typical of a chronic osteo-myelitis and identical to the lesion in the calcified substance of the jaws in connection with sinusless chronic dento-alveolar abscess, so-called dental granuloma. These observations should be verified by means of microscopical sections of the tissues involved, a work which will consume considerable time and upon which we hope to be able to report at a future date.

If our conception of radiographic interpretation in connection with other pathologic conditions in the jaws is at all correct, and we believe it is, having been convincingly substantiated by clinical and laboratory methods, then the radiographic findings in connection with fixed bridgework are of considerable significance in their bearing upon clinical problems. In the series of cases radiographically examined, those cases in which the extractions had been performed only a few years previously were not considered.

MIXING AND PACKING AMALGAM. All high-grade alloys amalgamate rather slowly. It requires thorough rubbing of the particles in the presence of mercury to develop thorough amalgamation, conditions that prohibit a hand mix. Mixing is best accomplished by rapid rubbing in a deep mortar for a minimum time of three minutes. A measure of time is the only dependable guide to a thorough mix.

Thorough mixing is necessary to attain that plasticity most favorable to adaptation. It is also necessary to the greatest strength of the filling, and it is the means by which we may practically eliminate that movement immediately following the placing of the filling. This early movement is common to all alloys.

Mercury may be added or expressed during the mixing to meet the requirements stated.

### *Plasticity*

In all average large and proximo-occlusal cavities the amalgam mass must be extremely plastic during the filling of the first two-thirds of the cavity to insure adaptation in the use of high-grade alloys.

This important requirement is best accomplished by retaining sufficient of the excess mercury (commonly removed during the final mixing or kneading), to be removed after it has served its purpose, by thorough, forceful and orderly packing.

The excess mercury generally removed during the final mixing is not dangerous to the stability of the filling because this mercury is a surplus always removed by the very ordinary packing force.

The excess mercury that requires our special attention is the final excess. The complete removal of this dangerous excess is absolutely and solely dependent upon thorough, forceful and orderly condensation.

Decided plasticity and the importance of uniform condensation are two operative details, not previously recognized by the profession as essential to results, that must be accepted if we would make, with reasonable uniformity, non-leaking and stable amalgam fillings.

*Tamping*

Was designed and has proved to be an aid to uniform condensation. Tamping is done by lightly tapping each piece of amalgam added with the plugger until it spreads as a uniformly thick, dense, and plastic layer in contact with the surrounding walls, and well into the angles, then we may proceed to condense. This procedure provides uniform bulk and plasticity under each thrust of the plugger. Experience shows a material improvement in adaptation when this detail is carried out.

*Packing*

The packing should be as forcible and thorough as possible and applied with the greatest possible uniformity. The most practical means of accomplishing this in the mouth is by an orderly stepping of the plugger.

Following the detail of tamping, we start the packing of each piece of amalgam with a medium or large plugger at the center of the filling, stepping a little less than its diameter in one direction in a gradually enlarging circle until the cavity walls are reached. The final condensation of each piece of amalgam is done by stepping the cavity walls.

To step the cavity walls we use the smallest plugger (size about twelve by eighteen tenths of a millimeter in diameter), stepping a little less than its long diameter immediately against the walls and well into the angles once around the cavity.

With an orderly packing, the excess mercury is always driven in one direction, outward, where it is at all times free to come to the surface to be removed as it appears.

Packing without order results in irregular condensation. Those parts of the filling thoroughly condensed will contain no excess mercury, and, as a result, will be abundantly strong, the parts of the filling less thoroughly condensed will contain excess mercury, and, as a result, the fillings will be weak and unstable with corresponding possible defects in adaptation.

With an orderly packing the final excess mercury finds its last lodgment immediately against the cavity walls and particularly in the angles, at which points it will exert its worst effects if allowed to remain. Forcibly stepping the cavity walls is the one procedure that will most certainly remove this dangerous excess and at the same time perfect the adaptation.

The operating details of condensation are the most exacting and difficult to acquire because they must be carried out simultaneously. To develop the skill necessary to uniform results, demands our most thoughtful consideration until correct habits have been formed.

As the filling approaches completion, the amalgam should be gradually less plastic, until at the finish, the excess mercury may be pinched out forcibly to the point where it still remains a workable mass.

The cavity should be filled with an excess, covering well all margins, after which the surface should be thoroughly condensed with the largest plugger. This final condensation may be done with pluggers sufficiently large to cover the whole filling, in combination with the heaviest possible mallet force; or the entire filling should be forcibly compressed with the thumb, holding the amalgam against all margins at the same time for a few moments. This will express the final excess mercury.

The filling should now remain undisturbed for about three minutes, after which it should be trimmed and carved to correct proximal and occlusal form, as far as this is possible; and the surface may be made smooth by light burnishing, and the use of soft brush wheels and pumice. To be given the final polish at a subsequent sitting.—Extract, *Journal N. D. A.*

